# Common analysis of direct RNA sequencinG CUrrently leads to misidentification of m^5^C at GCU motifs

**DOI:** 10.26508/lsa.202302201

**Published:** 2023-11-29

**Authors:** Kaylee J Watson, Robin E Bromley, Benjamin C Sparklin, Mark T Gasser, Tamanash Bhattacharya, Jarrett F Lebov, Tyonna Tyson, Nan Dai, Laura E Teigen, Karen T Graf, Jeremy M Foster, Michelle Michalski, Vincent M Bruno, Amelia RI Lindsey, Ivan R Corrêa, Richard W Hardy, Irene LG Newton, Julie C Dunning Hotopp

**Affiliations:** 1 Institute for Genome Sciences, University of Maryland School of Medicine, Baltimore, MD, USA; 2 Department of Biology, Indiana University , Bloomington, IN, USA; 3 New England Biolabs , Ipswich, MA, USA; 4 Department of Biology, University of Wisconsin Oshkosh , Oshkosh, WI, USA; 5 Department of Microbiology and Immunology, University of Maryland School of Medicine, Baltimore, MD, USA; 6 Greenebaum Cancer Center, University of Maryland School of Medicine, Baltimore, MD, USA

## Abstract

RNA modifications, such as methylation, can be detected with Oxford Nanopore Technologies direct RNA sequencing. One commonly used tool for detecting 5-methylcytosine (m^5^C) modifications is Tombo, which uses an “Alternative Model” to detect putative modifications from a single sample. We examined direct RNA sequencing data from diverse taxa including viruses, bacteria, fungi, and animals. The algorithm consistently identified a m^5^C at the central position of a GCU motif. However, it also identified a m^5^C in the same motif in fully unmodified in vitro transcribed RNA, suggesting that this is a frequent false prediction. In the absence of further validation, several published predictions of m^5^C in a GCU context should be reconsidered, including those from human coronavirus and human cerebral organoid samples.

## Abstract (precis)

Published predictions of 5-methylcytosine at GCU sites inferred from direct RNA sequencing and the Tombo Alternative Model should be reconsidered unless further validated with orthogonal methods.

## Introduction

**Figure 1.**
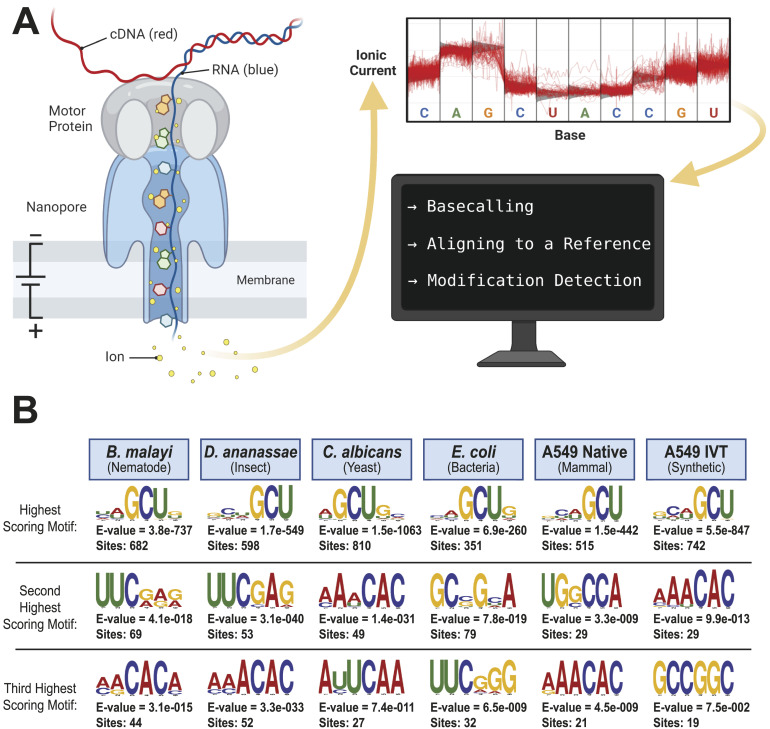
Oxford Nanopore Technologies direct RNA sequencing and GCU motif detection. **(A)** Schematic showing how RNA molecules are directly sequenced with Oxford Nanopore Technologies, followed by basecalling of the signal data produced by changes in ionic current, mapping to a reference, and detection of modified bases. Adapted from “Nanopore Sequencing,” by BioRender.com (2022). Retrieved from https://app.biorender.com/biorender-templates. **(B)** Three most significantly enriched MEME suite motifs in the 10-nucleotide sequences surrounding the top 1,000 putative modifications detected by the Tombo 5-methylcytosine Alternative Model for *B. malayi*, *D. ananassae*, *C. albicans*, *E. coli*, A549 native RNA, and A549 in vitro transcribed RNA. The top five motifs are shown in Table S3.

Oxford Nanopore Technologies (ONT) direct RNA sequencing (Fig 1A) enables detection of RNA modifications. A modified base produces an altered electrical current and/or dwell time relative to a canonical base that can be detected with algorithms (Garalde et al, 2018; Smith et al, 2019; Workman et al, 2019).

Tombo is commonly used and has three methods for detecting 5-methylcytosine (m^5^C) modifications, including two for detecting m^5^C in a single sample: “Alternative Model” and “de novo Model” (Stoiber et al, 2017 *Preprint*). The Alternative Model detects signals that fall within the expected range for a m^5^C modification. In contrast, the de novo detection method identifies differences from a canonical base model and is unable to predict the type of putative modification. Given that these methods can be run on a single sample, they have been used to predict modifications in many biological samples including *Arabidopsis thaliana* (Zhang et al, 2020), SARS-CoV-2 (Kim et al, 2020), human coronavirus 229E (Viehweger et al, 2019), and human cerebral organoid RNA (Bilinovich et al, 2020). Using the Alternative Model, coronavirus and the human cerebral organoid RNA are reported to have a modified GCU motif (Viehweger et al, 2019; Bilinovich et al, 2020).

The third Tombo method, “Sample Compare,” detects modifications by comparing the raw signals of two samples but is unable to predict the type of modification (Stoiber et al, 2017 *Preprint*). Sample Compare can detect differing levels of modification between two biological samples, or it can detect putative modifications in a single sample through comparisons with unmodified RNA generated by in vitro transcription (IVT) or knockout/knockdowns of modifying enzymes. However, this is limited by the ability to generate IVT RNAs or knockout/knockdowns. Sample Compare has been used to detect native SARS-CoV-2 modifications relative to an IVT control, but the Sample Compare predictions differed from those generated with the Alternative Model (Kim et al, 2020). Here, we identified a GCU motif that is a consistent false prediction of the Alternative Model across viral, bacterial, fungal, and animal RNA.

## Results

To broadly assess RNA modification patterns, RNA was isolated or existing data were used from diverse organisms including *Brugia malayi* FR3 (Animal: Nematode; 90 Mbp; 14,388 genes), *Drosophila ananassae* Hawaii (Animal: Insect; 220 Mbp; 23,553 genes), *Candida albicans* SC5314 (Fungi: Saccharomycetes; 15 Mbp; 6,271 genes), and *Escherichia coli* K-12 MG1655 (Bacteria: Gammaproteobacteria; 5 Mbp; 4,723 genes) (Blattner et al, 1997; Jones et al, 2004; Ghedin et al, 2007; Tvedte et al, 2021) (Table S1). The *B. malayi*, *D. ananassae*, and *C. albicans* libraries were prepared for ONT direct RNA sequencing with the standard protocol. Because ONT adapters require a poly(A) tail for annealing, *E. coli* total RNA was first polyadenylated using *E. coli* poly(A) polymerase before library preparation. This resulted in a larger proportion of ribosomal RNA present in the *E. coli* data (Table S1). Sequencing resulted in 822 Mbp *B. malayi*, 809 Mbp *D. ananassae*, 2.4 Gbp *C. albicans*, and 216 Kbp *E. coli* mapped reads (Table S1).

Table S1. Sequencing statistics for each RNA sample sequenced with Oxford Nanopore Technologies.

The Tombo Alternative Model detection method (Viehweger et al, 2019; Bilinovich et al, 2020; Kim et al, 2020; Zhang et al, 2020) was used to detect the cytosines that deviated from the canonical model and fell within the expected range for the m^5^C model. The methylated fraction of mapped reads that were predicted to have a m^5^C was identified at each cytosine and ranked from highest to lowest. The methylated fractions range from 0 to 1.0 across the entire transcriptome, so the number of m^5^C sites cannot be predicted as these results are not binary. The 1,000 most highly modified positions ranged between 0.85 and 1.0, meaning at least 85% of the reads at all 1,000 positions were predicted to be modified (Table S2).

Table S2. Statistics for the 10-nt sequences surrounding the top 1,000 5-methylcytosine fractions for each sample.

**Figure S1.**
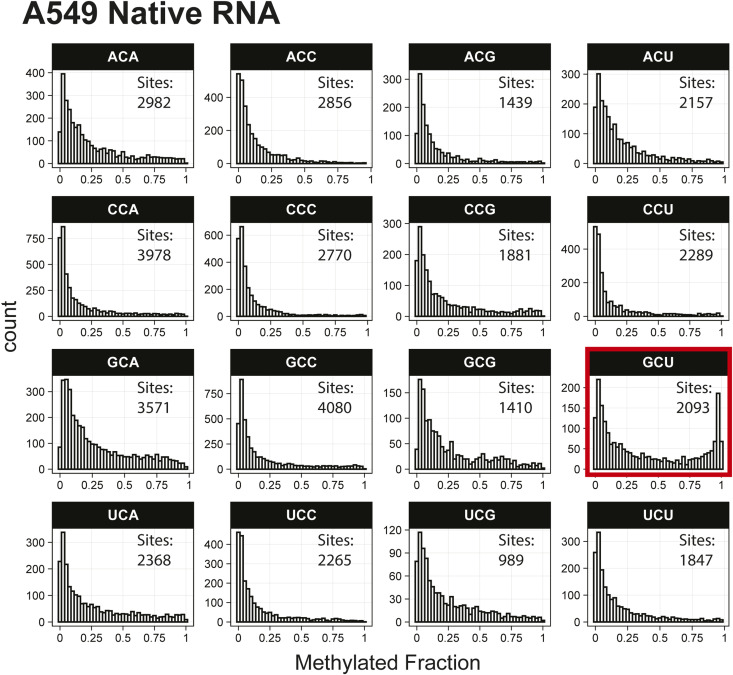
Histogram of A549 native RNA methylated fractions detected by the Tombo Alternative Model. Results plotted with R v4.0.3 using a bin width of 0.025.

**Figure S2.**
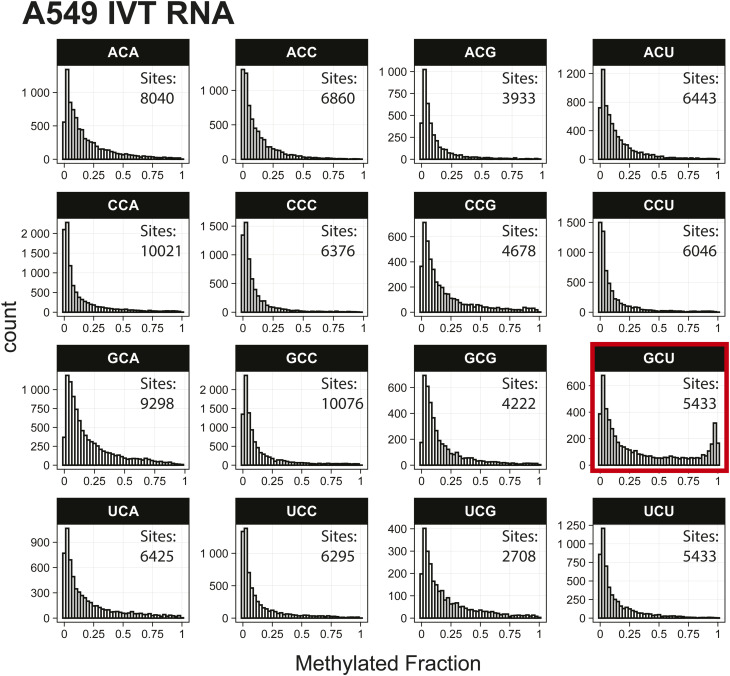
Histogram of A549 in vitro transcription RNA methylated fractions detected by the Tombo Alternative Model. Results plotted with R v4.0.3 using a bin width of 0.025.

**Figure S3.**
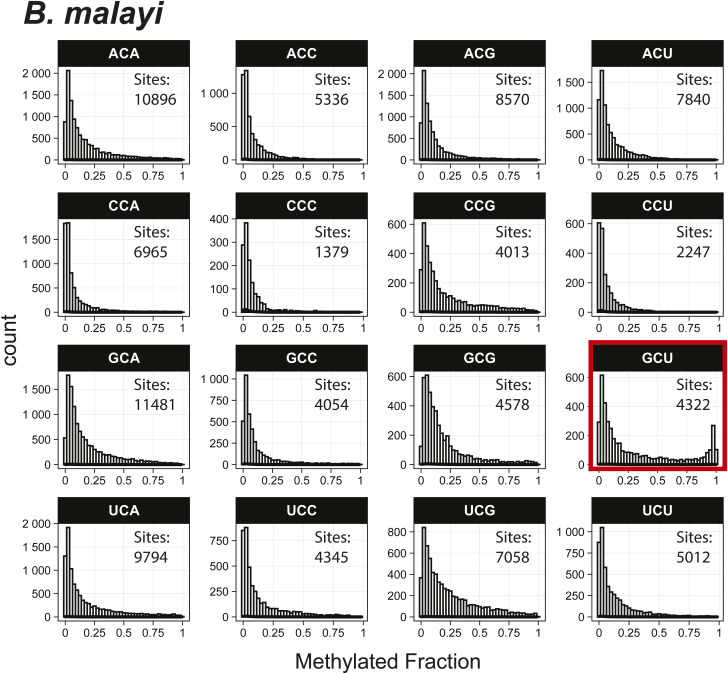
Histogram of *B. malayi* methylated fractions detected by the Tombo Alternative Model. Results plotted with R v4.0.3 using a bin width of 0.025.

**Figure S4.**
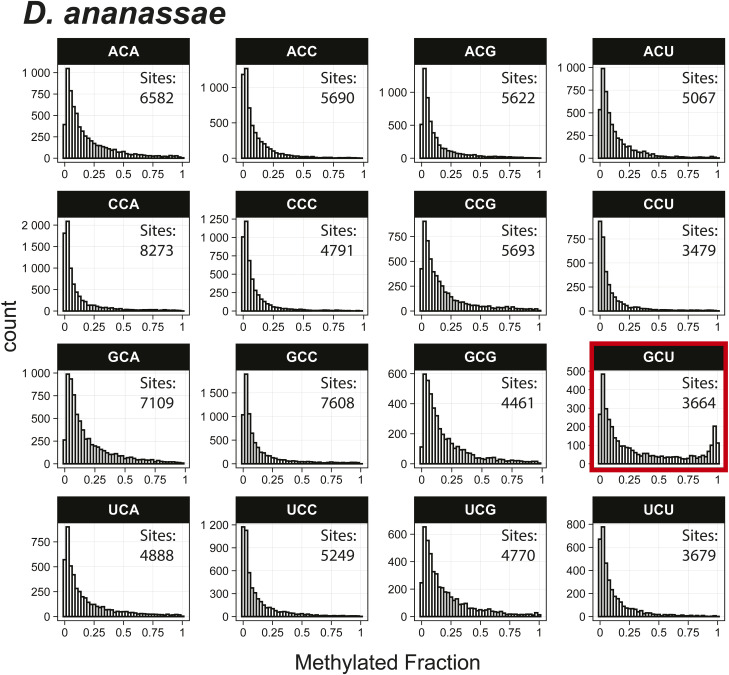
Histogram of *D. ananassae* methylated fractions detected by the Tombo Alternative Model. Results plotted with R v4.0.3 using a bin width of 0.025.

**Figure S5.**
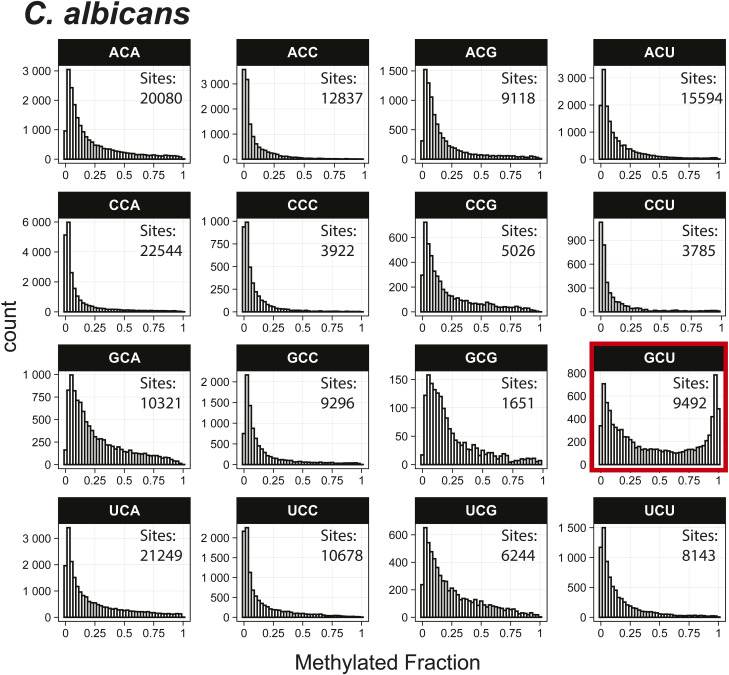
Histogram of *C. albicans* methylated fractions detected by the Tombo Alternative Model. Results plotted with R v4.0.3 using a bin width of 0.025.

**Figure S6.**
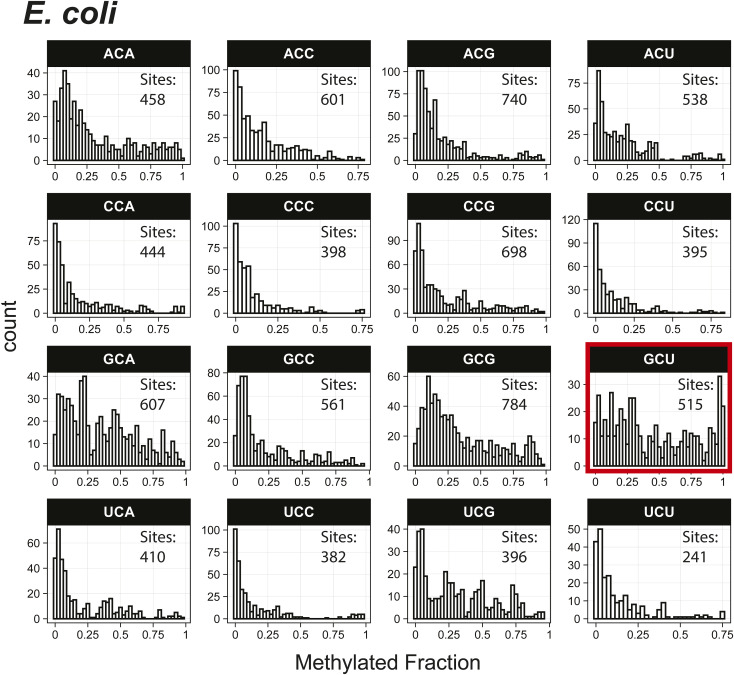
Histogram of *E. coli* methylated fractions detected by the Tombo Alternative Model. Results plotted with R v4.0.3 using a bin width of 0.025.

**Figure S7.**
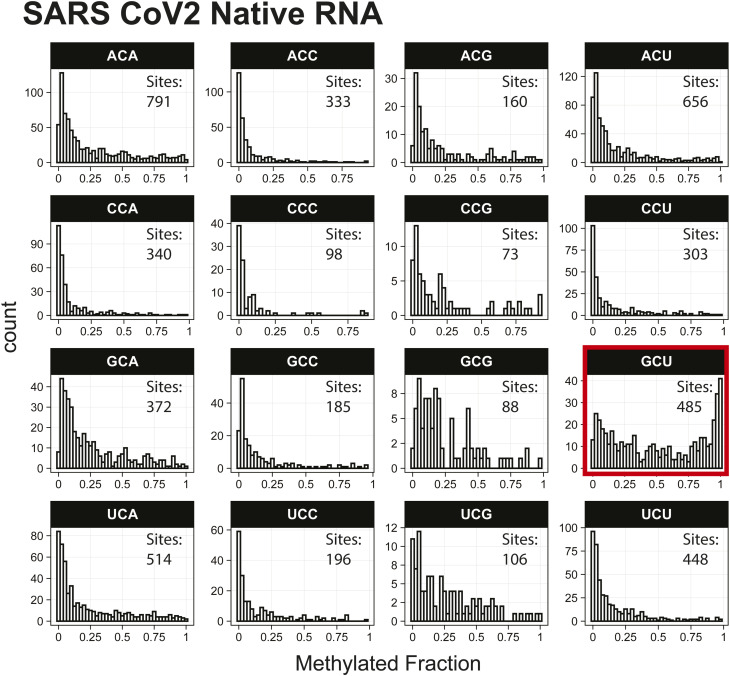
Histogram of SARS-CoV-2 native RNA methylated fractions detected by the Tombo Alternative Model. Results plotted with R v4.0.3 using a bin width of 0.025.

**Figure S8.**
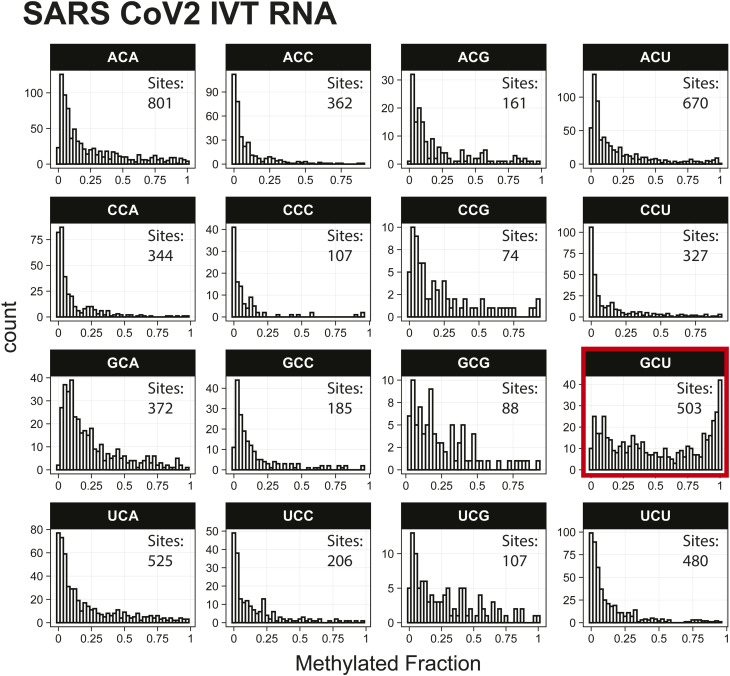
Histogram of SARS-CoV-2 in vitro transcription RNA methylated fractions detected by the Tombo Alternative Model. Results plotted with R v4.0.3 using a bin width of 0.025.

**Figure S9.**
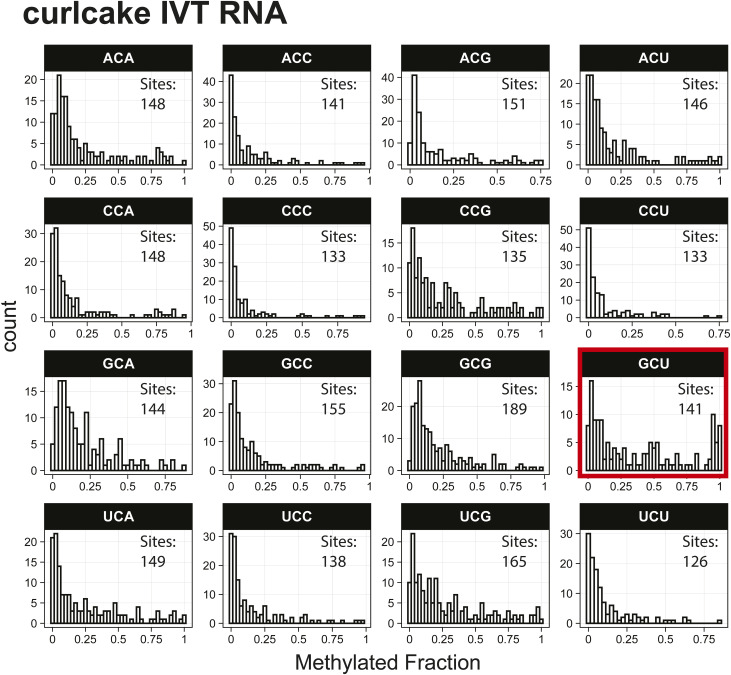
Histogram of curlcake synthetic sequence methylated fractions detected by the Tombo Alternative Model. Results plotted with R v4.0.3 using a bin width of 0.025.

Motifs associated with those cytosines that had the highest methylated fraction were detected using the MEME suite (Bailey et al, 2015) by inputting the 10-nucleotide sequences surrounding the predicted methylated cytosines and searching for enriched motifs between three and six nucleotides long. The most significant motif for all four organisms contained a GCU (Fig 1B) suggesting either an artifact or a m^5^C motif that spans multiple kingdoms of life. The latter would be similar to the DRACH motif observed for *N*6-methyladenosine (Harper et al, 1990; Dominissini et al, 2012; Schwartz et al, 2013; Parker et al, 2020). However, our analysis of the publicly available native A549 RNA (Chen et al, 2021 *Preprint*) and in vitro transcribed RNA from A549 cells (McCormick et al, 2023 *Preprint*) also indicated methylation of the GCU motif in both samples (Fig 1B). Given that the latter is in vitro transcribed and thus fully unmodified, this result suggests a high degree of false-positive predictions, particularly at GCU motifs. Several other motifs were frequently returned in the top 1,000 sequences analyzed with MEME, including UUC, AAACAC, GG, and CC (Table S3). Although the number of putative modified GCU sites varied by the organism, all organisms had over four times as many putative modified GCU sites as any other motif in the top 1,000 predicted modified positions. Compared to every other 3-mer with a central cytosine, GCU had higher methylated fractions across all samples analyzed, including in vitro transcribed SARS-CoV-2 RNA (Kim et al, 2020) and in vitro transcribed “curlcake” constructs that lack modified bases (Liu et al, 2019) (Figs S1, S2, S3, S4, S5, S6, S7, S8, and S9). SARS-CoV-2 native RNA and in vitro transcribed RNA are indistinguishable with respect to the frequency distribution of the methylated fraction at GCU motifs (Figs S7 and S8).

Table S3. Summary of all five motifs in the top 1,000 5-methylcytosine fractions. Motifs in gray boxes were not significantly enriched.

**Figure 2.**
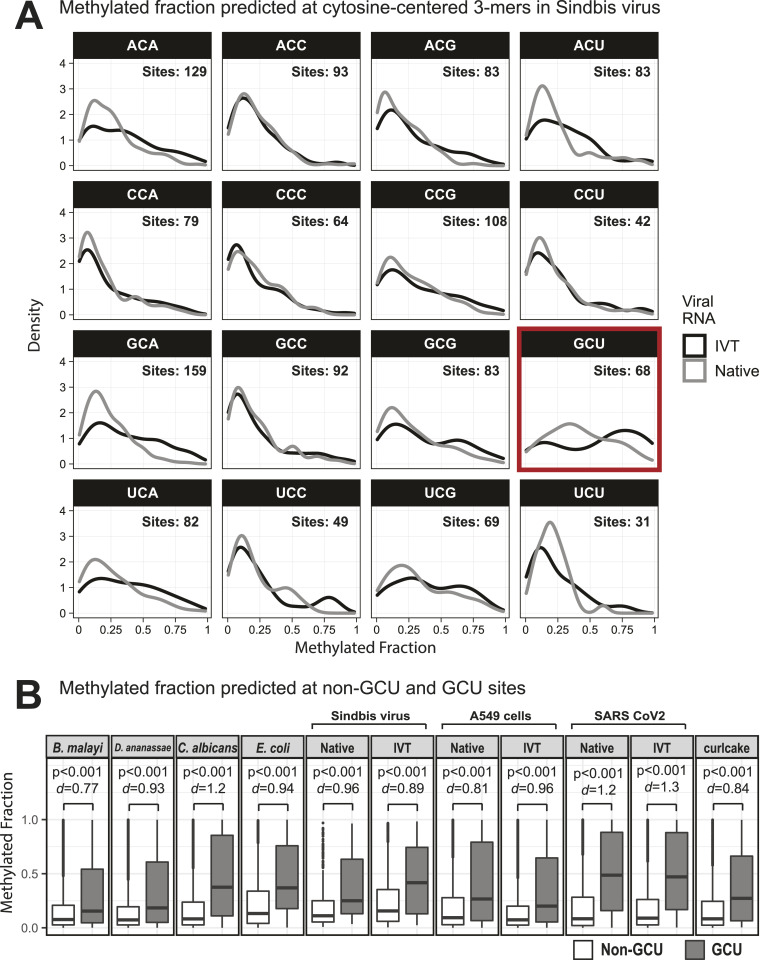
Methylated fractions predicted by the Tombo Alternative Model for 5-methylcytosine. **(A)** Density plots of the methylated fraction at all 3-mers containing a central cytosine in native and in vitro transcription viral RNA. Cytosine positions were filtered for depth >10 and methylated fraction >0 in both in vitro transcription and native samples. Histograms are available in Figs S10 and S11. **(B)** Boxplot showing distributions of the methylated fractions detected by the Tombo 5-methylcytosine Alternative Model. The methylated fraction was extracted for cytosines with a depth >100 except in the lower depth Sindbis virus samples, where cytosines with a depth >10 were retained. Methylated fractions were grouped based on the non-GCU and GCU sequence context. Statistical significance based on *P*-value from a two-tailed *Z* test and Cohen’s *d* effect size.

**Figure S10.**
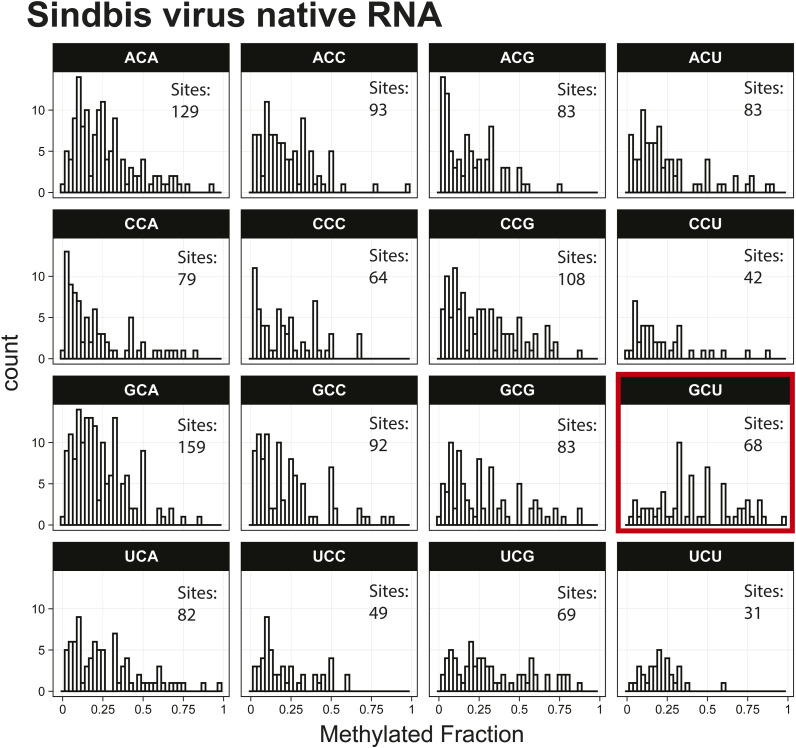
Histogram of Sindbis virus native RNA methylated fractions detected by the Tombo Alternative Model. Results plotted with R v4.0.3 using a bin width of 0.025.

**Figure S11.**
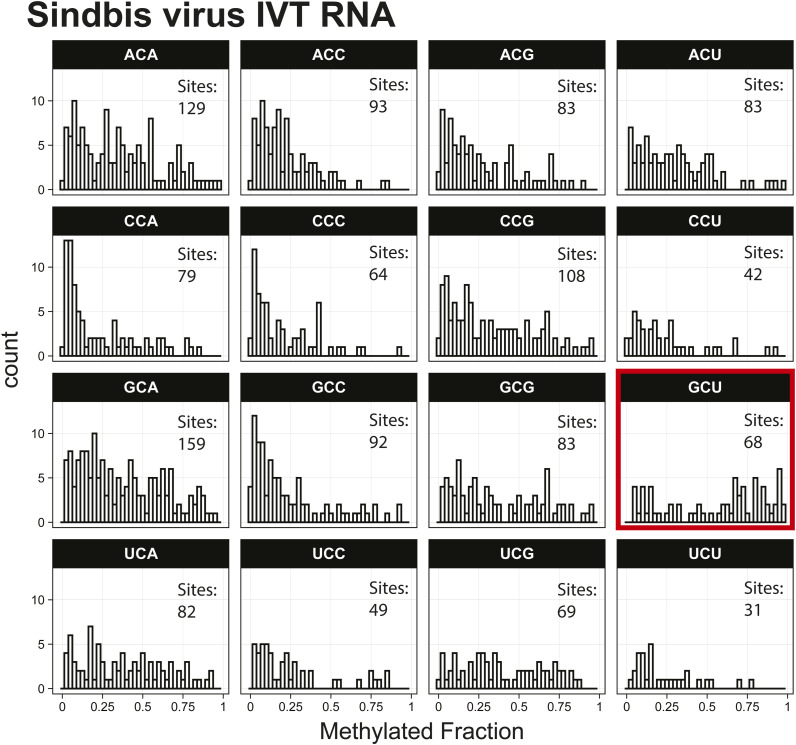
Histogram of Sindbis virus in vitro transcription RNA methylated fractions detected by the Tombo Alternative Model. Results plotted with R v4.0.3 using a bin width of 0.025.

To further support that erroneous predictions were occurring with the Tombo Alternative Model detection method, the 11.7-kbp Sindbis virus (SINV) RNA genome was in vitro transcribed and compared with native viral RNA from infected JW18 insect cells, which contains modified bases (Bhattacharya et al, 2017, 2020, 2021, 2022). At 11.7 kbp, the Sindbis virus RNA is easily prepared by IVT and is long enough to have enough GCUs to undertake the analysis. Liquid chromatography–tandem mass spectrometry (LC-MS/MS) confirmed the absence of modified bases in the in vitro transcribed SINV RNA (Table S4). Sequencing resulted in 320-kbp native SINV and 1.38 Mbp of IVT SINV sequence reads (Table S1), which were analyzed separately for m^5^C modifications using the Tombo Alternative Model. Because SINV has a more limited number of sites and only contains 146 GCU sites and 2,970 total cytosines, a top 1,000 putative modification site analysis is uninformative. Whereas in whole organisms, the lowest methylated fraction in the top 1,000 putative modified sites is 0.85, in the virus, the lowest methylated fraction is 0.1. Conversely, if we only use sites that are at least 85% methylated, we would only have 14 positions for a motif analysis, which is insufficient. Therefore, instead of the motif-based analysis of the top 1,000 putative modification sites, all 3-mers with a central cytosine in the viral genome were analyzed individually for the IVT and native SINV RNA. The methylated fraction in GCU motifs tended to be higher than any other 3-mer in the SINV RNA, in the IVT RNA, and also in all other samples examined (Figs 2A and S10 and S11). The median predicted methylated fraction for GCU sites was 1.2–2.7-fold higher relative to non-GCU context cytosines in *B. malayi*, *D. ananassae*, *C. albicans*, *E. coli*, SINV, A549 cells, and SARS-CoV-2, as well as multiple IVT-prepared RNA samples that are fully unmodified (Fig 2B).

Table S4. Quantification results from LC-MS/MS analysis of Sindbis virus in vitro transcription RNA.

## Discussion

The ability to detect RNA modifications in a single sample using a m^5^C-specific pretrained model has made the Tombo Alternative Model an attractive tool for epitranscriptomics analysis. Using this method, a GCU motif is consistently predicted to contain a central m^5^C in our data across diverse taxa and in data reported by others (Viehweger et al, 2019; Bilinovich et al, 2020). However, this GCU motif is also predicted to be methylated in fully unmodified IVT-prepared RNA. The detection of “modifications” in IVT RNA indicates this is merely an artifact of the detection algorithm. The GCU motif has been reported in numerous papers as a highly modified sequence, highlighting a need for improved methods and standards in RNA modification detection from direct RNA sequencing data. Without further experimental validation of GCU modification, results reporting GCU modifications using this analysis method should be considered carefully, including previously published results (Viehweger et al, 2019; Bilinovich et al, 2020). This problem likely extends to many other studies as well that report modifications but do not report what motifs or sites are modified. Epitranscriptomics predictions from a single sample using this analysis method without experimental validation should be critically evaluated or re-evaluated (e.g., Viehweger et al, 2019; Bilinovich et al, 2020; Kim et al, 2020; Zhang et al, 2020).

## Materials and Methods

### *Brugia malayi* RNA isolation

RNA was isolated from adult female *B. malayi* according to previously described methods (Chung et al, 2018). Briefly, third-stage larvae (L3) were isolated from mosquitoes infected with *B. malayi* according to the NIAID/NIH Filariasis Research Reagent Resource Center (FR3) Research Protocol 8.4 (www.filariasiscenter.org). The L3 larvae were injected into the peritoneal cavities of Mongolian gerbils (Charles River, Wilmington, MA, USA), and female adult worms were harvested from the peritoneal cavities after euthanizing the gerbils. The worms were washed in warm RPMI media containing 0.4 U penicillin and 4 μg streptomycin per ml, flash-frozen, and stored at −80°C. RNA was isolated using TRIzol (Zymo Research, Irvine, CA, USA) and homogenized in a TissueLyser (QIAGEN), followed by a PureLink RNA Mini column (Ambion). After isolation, RNA was treated with the TURBO DNA-free kit (Ambion; Thermo Fisher Scientific) according to the manufacturer’s protocol.

### *C. albicans* RNA isolation

A single colony of *C. albicans* strain SC5314 was grown overnight in YPD at 30°C at 225 rpm on an Excella E25R rotary shaker (New Brunswick Scientific). After RT centrifugation at 3,500 rpm using an SX4750 rotor in an Allegra X-14R centrifuge (Beckman Coulter) for 10 min, the cell pellet was resuspended in 1 ml TRIzol (Zymo Research, Irvine, CA, USA) reagent per 100-mg pellet. β-Mercaptoethanol was added at a ratio of 1:100, and the pellet was homogenized using a bead beater and TissueLyser (QIAGEN) at 30 s^−1^ for 8 min. After centrifugation at 12,000*g* for 5 min at 4°C, followed by incubation at RT for 5 min, 0.2 ml chloroform was added for every 1 ml TRIzol. The sample was shaken by hand for 15 s, incubated at RT for 3 min, loaded into a prespun Phase Lock Gel heavy tube (5Prime), and centrifuged at 12,000*g* for 5 min at 4°C. The upper phase, along with 1 vol of 100% ethanol, was loaded onto a PureLink RNA Mini column (Ambion). The remaining steps were performed according to the Pure Link RNA Mini Kit protocol, and the RNA was eluted in 30 μl RNase-free water and stored at −80°C. Before sequencing, poly(A) selection was performed according to the Dynabeads mRNA Purification Kit protocol (Thermo Fisher Scientific).

### *E. coli* RNA isolation

To create a starter culture, a single colony of *E. coli* K12 MG1655 was grown overnight in LB broth at 37°C at 200 rpm on an Excella E25R rotary shaker (New Brunswick Scientific). The overnight culture was diluted 1:100 in fresh LB broth, incubated at 37°C with aeration at 200 rpm on an Excella E25R rotary shaker (New Brunswick Scientific), and harvested at OD_600_ 0.50. RNA was isolated from pelleted cells using RNeasy Mini Kit (QIAGEN) with QIAGEN RNAprotect Bacteria Reagent and on-column DNase digestion following the manufacturer’s instructions.

### SINV

SINV stocks were generated as previously described (Lindsey et al, 2021). Briefly, baby hamster kidney fibroblasts (BHK-21 cells) were transfected with 1 μg in vitro transcribed SINV TE12 viral RNA (described below) to generate a virus stock that was used to infect new BHK-21 cells. The supernatant from the new BHK-21 cells was collected, purified, and used to infect *Wolbachia* (strain *w*Mel) colonized, *Drosophila melanogaster*–derived JW18 cells grown in serum-free Shields and Sang media at MOI of 10. Infected JW18 cells were harvested 5 d post-infection, and total RNA was extracted using TRIzol reagent (Invitrogen), followed by RQ1 RNase-free DNase (NEB) treatment using the manufacturer’s protocol.

### IVT

IVT was carried out using the TE12 BC 4.10 plasmid received from the Hardy Lab at Indiana University Bloomington. The pGEM-based plasmid contains a previously described SINV TE12 clone (Bailey et al, 2015) with the addition of an SP6 promoter for IVT and an 8-nucleotide barcode inserted +7 into the 3′ UTR. 1 μg plasmid was linearized with XhoI (Promega Corp.) at 37°C for 1.5 h. The DNA was then precipitated using 1/20th vol 0.5 M EDTA, 1/10th vol 5 M NH_4_ acetate, and 2 vol ethanol, followed by chilling at −20°C for 15 min. The DNA was pelleted for 15 min at max speed, and the supernatant was removed, followed by resuspension in 2 μl water. SINV RNA was in vitro transcribed from the linearized DNA template according to the Invitrogen MEGAscript SP6 Kit (Thermo Fisher Scientific Inc.) protocol with an incubation time of 2.5 h and addition of 2 μl m^7^G RNA cap analog (NEB), followed by precipitation with the Lithium Chloride Precipitation Solution provided in MEGAscript SP6 Kit.

### Polyadenylation of *E. coli* RNA

Poly(A) tailing of isolated *E. coli* K12 RNA was performed using *E. coli* poly(A) polymerase (NEB). The reaction was incubated for 30 min at 37°C and stopped by the addition of EDTA to a 10 mM final concentration.

### ONT library preparation and sequencing

For all samples, RNA was quantified and assessed for quality on DeNovix DS-11 Spectrophotometer (DeNovix), a Qubit fluorometer using the Qubit RNA High Sensitivity kit (QIAGEN), and/or Agilent TapeStation (Agilent). SQK-RNA002 Direct RNA Sequencing Kit (ONT) was used for library preparation, and sequencing was performed using ONT MinION with an R9 flow cell. Reads were basecalled using Guppy v6.4.2 with the “rna_r9.4.1_70bps_hac.cfg” model and filtered using a minimum quality score cutoff of 7.

### Direct RNA sequencing data reuse

Publicly available direct RNA sequencing data were downloaded from the NCBI Sequence Read Archive database for *D. ananassae* (SRR15923920; Tvedte et al, 2022), A549 IVT RNA (SRR23950397; McCormick et al, 2023 *Preprint*]), and curlcake synthetic sequences (SRR8767350; Liu et al, 2019). A549 native RNA sequencing data were downloaded from the Singapore Nanopore Expression Project (https://registry.opendata.aws/sgnex/) using the SGNex_A549_directRNA_replicate1_run1 dataset (Chen et al, 2021 *Preprint*). SARS-CoV-2 native and IVT RNAs (Kim et al, 2020) were downloaded from Open Science Framework (https://osf.io/8f6n9/).

### Analysis of sequencing data

Because Tombo requires a transcriptome reference for analysis of RNA, reads were aligned with minimap2 v2.24 (Li, 2018) to *B. malayi* (GenBank: GCF_000002995.4), *D. ananassae* (GenBank: GCA_017639315.2), *C. albicans* (GenBank: GCA_000182965.3), SARS-CoV-2 (GenBank: NC_045512.2), and human (GenBank: GCF_000001405.40) reference transcriptomes. The published curlcake sequences (Liu et al, 2019) were used as the curlcake reference. An *E. coli* reference transcriptome was produced from the *E. coli* genomic reference (GenBank: GCF_000005845.2) using the BEDOPS suite v2.4.36 (Neph et al, 2012) “gff2bed” tool to covert the GFF file to BED format, keeping all lines with “gene” as the description for the “region” column, and filtering the reference genome with the resulting BED file using the BEDTools suite v2.27.1 (Quinlan & Hall, 2010) “getfasta” tool. The SINV TE12 reference sequence was sent by the Hardy Lab at Indiana University Bloomington.

The number of reads sequenced and mapped was determined for each sample using SAMtools v1.17 (Danecek et al, 2021) with the “-F 2308” filter applied to BAM files. The SeqKit v2.0.0 (Shen et al, 2016) “stats” tool was used to calculate the N50, bases sequenced, and bases mapped. For more accurate values, regions that were “soft-clipped” during mapping were removed with JVarkit (https://figshare.com/articles/journal_contribution/JVarkit_java_based_utilities_for_Bioinformatics/1425030), and the BAM file was converted to FASTQ format with “samtools fastq.” To determine reads mapped to rRNA, GFF files for each organism were filtered to include only rRNA regions. The BEDOPS suite “gff2bed” tool was used to create a BED file from the filtered GFF file, and rRNA reads were pulled from the BAM file after mapping to the reference genome with SAMtools view, using the “-L” option and the BED file containing rRNA regions.

### RNA modification detection and motif discovery

m^5^C RNA modifications were detected using the Tombo Alternative Model. Multi-read fast5 files were converted to single-read fast5 files using ONT fast5 API software and reannotated with Tombo preprocess annotate_raw_with_fastqs. The raw signal was then assigned to each base using Tombo resquiggle with the --rna option, and modified bases were detected with Tombo detect_modifications alternative_model.

### Motif discovery

Using Tombo, the 10-nt regions surrounding each of the top 1,000 modified cytosines were obtained with the “tombo text_output signif_sequence_context” tool with options “--num-regions 1,000 and --num-bases 10” specified. The output was a FASTA file of the 1,000 regions, and this was analyzed with MEME suite v5.5.1 (Bailey et al, 2015) using the following options: five motifs, 0-order model, min width = 3, max width = 6, and search given strand only. Results were plotted with R v4.0.3.

### LC-MS/MS

SINV IVT RNA (650 ng) was digested overnight to single nucleosides using Nucleoside Digestion Mix (NEB). LC-MS/MS analysis was performed by injecting digested RNA on Agilent 1290 Infinity II UHPLC equipped with a G7117A diode array detector and a 6495C triple quadrupole mass detector operating in the positive electrospray ionization mode (+ESI). UHPLC was carried out on a Waters XSelect HSS T3 XP column (2.1 × 100 mm, 2.5 μm) with a gradient mobile phase consisting of methanol and 10 mM aqueous ammonium acetate (pH 4.5). MS data acquisition was performed in the dynamic multiple reaction monitoring mode. Each nucleoside was identified in the extracted chromatogram associated with its specific MS/MS transition: rC [M+H]+ at m/z 244.1→112.1, rU [M+H]+ at m/z 245.1→113, rG [M+H]+ at m/z 284.1→152.1, rA [M+H]+ at m/z 268.1→136.1, Ψ [M+H]+ at m/z 245.1→209, m^5^C [M+H]+ at m/z 258.1→126.1, and m^6^A [M+H]+ at m/z 282.1→150.1. External calibration curves with known amounts of the nucleosides were used to calculate their ratios within the sample.

## Data Availability

All raw and processed sequencing data from this publication have been deposited in the NCBI Sequence Read Archive database (https://www.ncbi.nlm.nih.gov/sra) and assigned the BioProject identifier PRJNA944578 (Table S1). All commands and scripts are available on GitHub: https://github.com/Dunning-Hotopp-Lab/Common-Analysis-of-Direct-RNA-Sequencing-Misidentification-of-5mC-Modifications-at-GCU-Motifs.

## Supplementary Material

Reviewer comments

## References

[bib1] Bailey TL, Johnson J, Grant CE, Noble WS (2015) The meme suite. Nucleic Acids Res 43: W39–W49. 10.1093/nar/gkv416 25953851 PMC4489269

[bib2] Bhattacharya T, Newton ILG, Hardy RW (2017) Wolbachia elevates host methyltransferase expression to block an RNA virus early during infection. PLoS Pathog 13: e1006427. 10.1371/journal.ppat.1006427 28617844 PMC5472326

[bib3] Bhattacharya T, Newton ILG, Hardy RW (2020) Viral RNA is a target for wolbachia-mediated pathogen blocking. PLoS Pathog 16: e1008513. 10.1371/journal.ppat.1008513 32555677 PMC7326284

[bib4] Bhattacharya T, Rice DW, Crawford JM, Hardy RW, Newton ILG (2021) Evidence of adaptive evolution in wolbachia-regulated gene DNMT2 and its role in the dipteran immune response and pathogen blocking. Viruses 13: 1464. 10.3390/v13081464 34452330 PMC8402854

[bib5] Bhattacharya T, Yan L, Crawford JM, Zaher H, Newton ILG, Hardy RW (2022) Differential viral RNA methylation contributes to pathogen blocking in wolbachia-colonized arthropods. PLoS Pathog 18: e1010393. 10.1371/journal.ppat.1010393 35294495 PMC8959158

[bib6] Bilinovich SM, Uhl KL, Lewis K, Soehnlen X, Williams M, Vogt D, Prokop JW, Campbell DB (2020) Integrated RNA sequencing reveals epigenetic impacts of diesel particulate matter exposure in human cerebral organoids. Dev Neurosci 42: 195–207. 10.1159/000513536 33657557 PMC7990702

[bib7] Blattner FR, Plunkett G 3rd, Bloch CA, Perna NT, Burland V, Riley M, Collado-Vides J, Glasner JD, Rode CK, Mayhew GF, (1997) The complete genome sequence of Escherichia coli k-12. Science 277: 1453–1462. 10.1126/science.277.5331.1453 9278503

[bib8] Chen Y, Davidson NM, Wan YK, Patel H, Yao F, Low HM, Hendra C, Watten L, Sim A, Sawyer C, (2021) A systematic benchmark of nanopore long read RNA sequencing for transcript level analysis in human cell lines. *BioRxiv*. 10.1101/2021.04.21.440736 (Preprint posted April 22, 2021). PMC11978509 40082608

[bib9] Chung M, Teigen L, Liu H, Libro S, Shetty A, Kumar N, Zhao X, Bromley RE, Tallon LJ, Sadzewicz L, (2018) Targeted enrichment outperforms other enrichment techniques and enables more multi-species RNA-seq analyses. Sci Rep 8: 13377. 10.1038/s41598-018-31420-7 30190541 PMC6127098

[bib10] Danecek P, Bonfield JK, Liddle J, Marshall J, Ohan V, Pollard MO, Whitwham A, Keane T, McCarthy SA, Davies RM, (2021) Twelve years of samtools and bcftools. Gigascience 10: giab008. 10.1093/gigascience/giab008 33590861 PMC7931819

[bib11] Dominissini D, Moshitch-Moshkovitz S, Schwartz S, Salmon-Divon M, Ungar L, Osenberg S, Cesarkas K, Jacob-Hirsch J, Amariglio N, Kupiec M, (2012) Topology of the human and mouse m6a RNA methylomes revealed by m6a-seq. Nature 485: 201–206. 10.1038/nature11112 22575960

[bib12] Garalde DR, Snell EA, Jachimowicz D, Sipos B, Lloyd JH, Bruce M, Pantic N, Admassu T, James P, Warland A, (2018) Highly parallel direct RNA sequencing on an array of nanopores. Nat Methods 15: 201–206. 10.1038/nmeth.4577 29334379

[bib13] Ghedin E, Wang S, Spiro D, Caler E, Zhao Q, Crabtree J, Allen JE, Delcher AL, Guiliano DB, Miranda-Saavedra D, (2007) Draft genome of the filarial nematode parasite brugia malayi. Science 317: 1756–1760. 10.1126/science.1145406 17885136 PMC2613796

[bib14] Harper JE, Miceli SM, Roberts RJ, Manley JL (1990) Sequence specificity of the human MRNA n6-adenosine methylase in vitro. Nucleic Acids Res 18: 5735–5741. 10.1093/nar/18.19.5735 2216767 PMC332308

[bib15] Jones T, Federspiel NA, Chibana H, Dungan J, Kalman S, Magee BB, Newport G, Thorstenson YR, Agabian N, Magee PT, (2004) The diploid genome sequence of candida albicans. Proc Natl Acad Sci U S A 101: 7329–7334. 10.1073/pnas.0401648101 15123810 PMC409918

[bib16] Kim D, Lee JY, Yang JS, Kim JW, Kim VN, Chang H (2020) The architecture of SARS-CoV-2 transcriptome. Cell 181: 914–921.e10. 10.1016/j.cell.2020.04.011 32330414 PMC7179501

[bib17] Li H (2018) Minimap2: Pairwise alignment for nucleotide sequences. Bioinformatics 34: 3094–3100. 10.1093/bioinformatics/bty191 29750242 PMC6137996

[bib18] Lindsey ARI, Bhattacharya T, Hardy RW, Newton ILG (2021) Wolbachia and virus alter the host transcriptome at the interface of nucleotide metabolism pathways. mBio 12: e034722-20. 10.1128/mBio.03472-20 PMC7885120 33563832

[bib19] Liu H, Begik O, Lucas MC, Ramirez JM, Mason CE, Wiener D, Schwartz S, Mattick JS, Smith MA, Novoa EM (2019) Accurate detection of m(6)a rna modifications in native RNA sequences. Nat Commun 10: 4079. 10.1038/s41467-019-11713-9 31501426 PMC6734003

[bib20] McCormick CA, Akeson S, Tavakoli S, Bloch D, Klink IN, Jain M, Rouhanifard SH (2023) Multicellular, ivt-derived, unmodified human transcriptome for nanopore direct RNA analysis. *BioRxiv*. 10.1101/2023.04.06.535889 (Preprint posted April 6, 2023). PMC11221353 38962390

[bib21] Neph S, Kuehn MS, Reynolds AP, Haugen E, Thurman RE, Johnson AK, Rynes E, Maurano MT, Vierstra J, Thomas S, (2012) Bedops: High-performance genomic feature operations. Bioinformatics 28: 1919–1920. 10.1093/bioinformatics/bts277 22576172 PMC3389768

[bib22] Parker MT, Knop K, Sherwood AV, Schurch NJ, Mackinnon K, Gould PD, Hall AJ, Barton GJ, Simpson GG (2020) Nanopore direct RNA sequencing maps the complexity of arabidopsis MRNA processing and m(6)a modification. Elife 9: e49658. 10.7554/eLife.49658 31931956 PMC6959997

[bib23] Quinlan AR, Hall IM (2010) Bedtools: A flexible suite of utilities for comparing genomic features. Bioinformatics 26: 841–842. 10.1093/bioinformatics/btq033 20110278 PMC2832824

[bib24] Schwartz S, Agarwala SD, Mumbach MR, Jovanovic M, Mertins P, Shishkin A, Tabach Y, Mikkelsen TS, Satija R, Ruvkun G, (2013) High-resolution mapping reveals a conserved, widespread, dynamic MRNA methylation program in yeast meiosis. Cell 155: 1409–1421. 10.1016/j.cell.2013.10.047 24269006 PMC3956118

[bib25] Shen W, Le S, Li Y, Hu F (2016) Seqkit: A cross-platform and ultrafast toolkit for fasta/q file manipulation. PLoS One 11: e0163962. 10.1371/journal.pone.0163962 27706213 PMC5051824

[bib26] Smith AM, Jain M, Mulroney L, Garalde DR, Akeson M (2019) Reading canonical and modified nucleobases in 16s ribosomal RNA using nanopore native RNA sequencing. PLoS One 14: e0216709. 10.1371/journal.pone.0216709 31095620 PMC6522004

[bib27] Stoiber M, Quick J, Egan R, Eun Lee J, Celniker S, Neely RK, Loman N, Pennacchio LA, Brown J (2017) *De novo* identification of DNA modifications enabled by genome-guided nanopore signal processing. *BioRxiv*. 10.1101/094672 (Preprint posted April 10, 2017).

[bib28] Tvedte ES, Gasser M, Sparklin BC, Michalski J, Hjelmen CE, Johnston JS, Zhao X, Bromley R, Tallon LJ, Sadzewicz L, (2021) Comparison of long-read sequencing technologies in interrogating bacteria and fly genomes. G3 (Bethesda) 11: jkab083. 10.1093/g3journal/jkab083 33768248 PMC8495745

[bib29] Tvedte ES, Gasser M, Zhao X, Tallon LJ, Sadzewicz L, Bromley RE, Chung M, Mattick J, Sparklin BC, Dunning Hotopp JC (2022) Accumulation of endosymbiont genomes in an insect autosome followed by endosymbiont replacement. Curr Biol 32: 2786–2795.e5. 10.1016/j.cub.2022.05.024 35671755 PMC9311232

[bib30] Viehweger A, Krautwurst S, Lamkiewicz K, Madhugiri R, Ziebuhr J, Hölzer M, Marz M (2019) Direct RNA nanopore sequencing of full-length coronavirus genomes provides novel insights into structural variants and enables modification analysis. Genome Res 29: 1545–1554. 10.1101/gr.247064.118 31439691 PMC6724671

[bib31] Workman RE, Tang AD, Tang PS, Jain M, Tyson JR, Razaghi R, Zuzarte PC, Gilpatrick T, Payne A, Quick J, (2019) Nanopore native RNA sequencing of a human poly(a) transcriptome. Nat Methods 16: 1297–1305. 10.1038/s41592-019-0617-2 31740818 PMC7768885

[bib32] Zhang S, Li R, Zhang L, Chen S, Xie M, Yang L, Xia Y, Foyer CH, Zhao Z, Lam HM (2020) New insights into arabidopsis transcriptome complexity revealed by direct sequencing of native RNAs. Nucleic Acids Res 48: 7700–7711. 10.1093/nar/gkaa588 32652016 PMC7430643

